# *In Situ* Characterization of Intrahepatic Non-Parenchymal Cells in PSC Reveals Phenotypic Patterns Associated with Disease Severity

**DOI:** 10.1371/journal.pone.0105375

**Published:** 2014-08-20

**Authors:** Lena Berglin, Annika Bergquist, Helene Johansson, Hans Glaumann, Carl Jorns, Sebastian Lunemann, Heiner Wedemeyer, Ewa C. Ellis, Niklas K. Björkström

**Affiliations:** 1 Liver Immunology Laboratory, Unit of Gastroenterology and Hepatology, Department of Medicine, Karolinska Institutet, Karolinska University Hospital Huddinge, Stockholm, Sweden; 2 Division of Transplantation Surgery, Department of Clinical Science, Intervention and Technology, Karolinska Institutet, Karolinska University Hospital, Stockholm, Sweden; 3 Department of Medicine, Clinical Pathology and Cytology, Karolinska University Hospital, Stockholm, Sweden; 4 Department of Gastroenterology, Hepatology and Endocrinology, Hannover Medical School, Hannover, Germany; 5 Center for Infectious Medicine, Department of Medicine, Karolinska Institutet, Karolinska University Hospital Huddinge, Stockholm, Sweden; University of Basque Country, Spain

## Abstract

Liver-infiltrating T cells have been implicated in the pathogenesis of primary sclerosing cholangitis (PSC), however little information is available about changes in other cellular compartments in the liver during PSC. This study aimed to characterize non-parenchymal intrahepatic cells in PSC livers and to find associations between phenotypes and disease severity. Using immunohistochemistry, followed by automated image analysis and quantification and a principal component analysis, we have studied non-parenchymal intrahepatic cells in PSC-patient livers (n = 17) and controls (n = 17). We observed a significant increase of T cells in the PSC patients, localized to the fibrotic areas. MAIT cells, normally present at high numbers in the liver, were not increased to the same extent. PSC patients had lower expression of MHC class I than controls. However, the levels of NKp46+ NK cells were similar between patients and controls, nevertheless, NKp46 was identified as a phenotypic marker that distinguished PSC patients with mild from those with severe fibrosis. Beyond that, a group of PSC patients had lost expression of Caldesmon and this was associated with more extensive bile duct proliferation and higher numbers of T cells. Our data reveals phenotypic patterns in PSC patients associated with disease severity.

## Introduction

Primary sclerosing cholangitis (PSC) is a chronic liver disease with unknown etiology and incompletely understood pathogenesis. Like other cholangiopathies, PSC is characterized by chronic inflammation that leads to cholestasis, proliferation of cholangiocytes, fibrosis, and ductopenia [1]. During disease, cholangiocyte function is affected by proinflammatory mediators in the microenvironment, and in turn contribute to the activation of hepatic stellate cells (HSCs) [2]. HSCs may respond to the inflammation and transdifferentiate into profibrogenic myofibroblasts. Other cells in the portal tract, such as smooth muscle cells, may contribute to the repair mechanisms leading to fibrosis. In addition to their function as collagen-producing and fibrosis-generating cells, myofibroblasts secrete several cytokines, chemokines and other soluble factors that together modulate the immune response [2].

Although the pathogenesis is not fully understood, genetic associations support the theory that PSC is immune-mediated. The strongest genetic risk factors are located in the major histocompatibility complex (MHC) [3]. However, several other immune related genes, located in non-MHC loci, have been found to associate with PSC [4]. Examples include *CD28* and *CD226* [4], whose encoded receptors provide co-stimulatory signals for T cells and NK cells respectively [5], [6]. Thus, genetic studies suggest that components of both the adaptive and innate immunity are likely to take part in the pathogenesis of PSC.

T cells have been the focus of many studies on pathogenic mechanisms of PSC [7]. Clinical observations and the strong association with inflammatory bowel disease suggests that the immune system responds in a dysfunctional manner to bacterial stimuli [1]. For instance, PSC-patients have increased amounts of bacterial and fungal species in their bile and these have been shown to promote the appearance of high frequencies of peripheral blood Th17 and Th1/Th17 cells [8]. Furthermore, cytotoxic CD8 T cells, primed in the gut, might migrate to the liver and cause cholangitis when targeting the biliary epithelia [9]–[11].

Bile duct cells are known to be the target of the immune attack in PSC, but little is known about changes in other non-parenchymal cellular compartments in the liver during PSC [12]. In this project, the aim was to characterize and localize intrahepatic cells of both immune as well as non-immune cell origin in PSC livers to find distinct phenotypes and to investigate if these were associated with disease severity. Our data reveal heterogeneity within PSC-patients where individuals with certain phenotypic patterns have higher numbers of T cells and more extensive bile duct proliferation, indicative of disease activity.

## Materials and Methods

### Patients and liver tissue

Seventeen PSC-patients and 17 non-PSC controls were enrolled in this study. Sixteen of the PSC samples were obtained at the time of liver transplantation, and one sample was obtained from a patient undergoing liver-resection due to suspicion of cholangiocarcinoma, all at Karolinska University Hospital. PSC diagnosis was based on biochemical, clinical and cholangiographic features, typical history, and characteristic endoscopic and histologic findings. Control tissues were obtained from four donor livers from deceased individuals not used for liver transplantation because of technical reason, and from 13 patients undergoing liver resection due to suspicion of cancer (11 colorectal cancer metastasis, one leiomyosarcoma, and one neuroendocrine tumor). All control samples were derived from non-tumor affected areas of the resected part. Exclusion criteria for the non-PSC cohort included cholestatic disease or a primary malignancy in the liver. Informed written consent was obtained from all patients and the Regional Ethics Committee, Stockholm, Sweden, approved the study. Material from deceased liver donors were included in the study according to the regulations of the organ transplantation law of Sweden (1995∶831), that is, the donors prior written declaration was followed as well as the written informed consent from next of kin. Patient clinical characteristics are presented in Table 1. Upon collection, all liver specimens were embedded in Tissue-Tek OCT, snap-frozen, and stored at −80°C until sectioning

**Table 1:** Control and PSC-patient characteristics.

	Controls (n = 17)	PSC patients (n = 17)
Age, median (range)	58 (16–86)	36 (19–68)
Male	9 (53%)	10 (59%)
IBD	0^1^	14 (82%)
UC	NA	10 (71%)
CD	NA	4 (29%)
Ltx	0	16 (94%)
Dysplasia/cancer in explanted liver	NA	3 (19%)
Liver resection	13 (76%)	1 (6%)
Deceased donors	4 (24%)	NA
MELD score, mean ± SD	NA	13±8
Serum IgG (g/L), mean ± SD	NA	15.5±4.6
ALP (µkat/L), mean ± SD	1.8±1.0	5.4±4.4
Bilirubin (µmol/L), mean ± SD	7.9±4.9	89.2±166.1
ALT (µkat/L), mean ± SD	0.6±0.2	1.3±0.7
CRP (mg/L), mean ± SD	5.3±6.6	24.4±32.9
Urso dose (g/day), mean ± SD	NA	0.8±0.6
Fibrosis score, 0/1/2/3/4	12/5/0/0/0	0/4/0/0/13
Inflammation grade, 0/1/2/3/4	14/3/0/0/0	4/4/9/0/0
Bile duct proliferation stage, 0/1/2/3	3/14/0/0	3/2/7/5

Abbreviations: IBD = inflammatory bowel disease; UC = ulcerative colitis; CD = Crohńs disease; NA = not applicable, Ltx = liver transplantation, MELD = Model for End-Stage Liver Disease, S.D = standard deviation, ALP = alkaline phosphatase, ALT = alanine aminotransferase, CRP = C-reactive protein, Urso = ursodeoxycholic acid.

1 Information not available for four controls.

### Histological grading

For histological grading, liver sections were stained with hematoxylin and eosin, analyzed in a blinded fashion by an experienced liver pathologist, and scored according to a system evaluating inflammation (grade) and fibrosis (stage) on a non-continuous, 0–4 grade scale. Bile duct proliferation was evaluated according to a scoring system with; 0 = 1–2 bile ducts per portal zone, 1 = minimal proliferation, corresponding to 3–4 ducts per portal zone, 2 = moderate proliferation corresponding to 5–7 ducts per portal zone, 3 = intensive proliferation corresponding to 8 or more bile ducts per portal zone. The liver histology of the patients and controls is presented in Table 1.

### Antibodies

The following commercially available antibodies were used: anti-CK19 (clone: RCK108, 1∶500 dilution), anti-CK7 (OV-TL 12/30, 1∶5000), anti-Human Epithelial Antigen (BerEP4, 1∶1000), and anti-IgG1 (same dilution as primary antibody) as isotype, all from Dako. Anti-CD31 (JC/70A, 1∶1000) (Abcam), anti-Caldesmon (E89, 1∶3000) (Abcam), anti-CD3 epsilon (EP449E, 1∶100) (Epitomics), anti-TCR-Vα7.2 (3C10, 1∶100) (BioLegend), anti-NKp46 (195314, 1∶100) (R&D Systems), anti-CD163 (EDHu-1, 1∶2000) (AbD Serotec), anti-HLA-A,B,C (W6/32, 1∶400) (Biolegend), purified rabbit IgG (concentration matched to primary antibodies) (Epitomics), anti-IgG2A (1333304, 1∶400) (R&D Systems), anti-IgG2B (73009, 1∶100) (R&D Systems) as isotype controls.

### IHC method

5 µm tissue sections from frozen samples were placed on SuperFrost Ultra Plus slides (Histolab) and stored in –20 freezer until staining. Next, sections were air-dried 10 minutes and thereafter fixed in 4% paraformaldehyde (Sigma Aldrich) for 20 minutes on ice. Sections were incubated with Bloxall (Vector Laboratories) for 10 minutes and then blocked in Innovex background Buster (Innovex Biosciences) for 20 minutes at room temperature. Samples were incubated with primary antibodies over night in a moisture chamber at +4°C. Subsequently, sections were incubated with ImmPRESS mouse or rabbit secondary antibodies (Vector Laboratories) for 30 minutes at room temperature and specific staining was detected by incubation with ImmPACT DAB (Vector Laboratories). Finally, tissue sections were counterstained with Hematoxylin (Histolab) and mounted with Kaiserśs glycerol gelatine (Merck Millipore). Positive staining was visualized by light microscopy (Leica DM 4000B).

### IHC analysis

Immunohistochemically stained specimens were analyzed in a blinded fashion by acquired computerized image analysis (ACIA). Twenty consecutive photomicrographs were taken from each tissue section, which corresponded to the majority of the sample area. The results are presented both as ACIA-values, calculated as the percentage of the area that stained positively multiplied by the mean intensity of positive staining, and as frequency of expression, calculated as percentage of positively stained area divided by the total sample area.

### mRNA preparation and quantification

Total mRNA was isolated from liver with Trizol (Ambion Life technologies) using the manufacturers protocol and dissolved in 50 ul RNase free water. cDNA synthesis from 1 ug RNA was performed using Applied Biosystem’s High capacity cDNA reverse transcription kit. Quantification of mRNA was performed using TaqMan real time PCR, employing the Applied Biosystems 7500 Fast Real-Time PCR System. All samples were analyzed in triplicates. Relative mRNA expression was calculated from the Ct-values against the housekeeping gene *Cyclophilin A* using the comparative delta-Ct method. The following primers targeting specific mRNAs were used in the study: Hs00761767_s1 (*Cytokeratin19*), Hs00921982_m1 (*Caldesmon*), Hs01062241_m1 (*CD3*), Hs00183118_m1 (*NKp46*), Hs01058806_g1 (*HLA-A*), Hs00818803_g1 (*HLA-B*), and Hs99999904_m1 (*Cyclophilin A*).

### Statistics

Statistical analyzes were performed using Prism version 6.0 (GraphPad Software Inc). Kolmogorov-Smirnov tests were performed to probe if values were normally distributed. For comparisons of independent groups, the Students t test or the Mann Whitney *U* test were performed. For comparison of paired samples, a paired t test or Wilcoxon signed-rank test were used. One-way ANOVA with a Bonferroni post-analysis was used for multiple groups. For correlation analyzes, linear regressions were performed. Principal component analysis (PCA) was performed using Qlucore Omics Explorer v2.2 (Qlucore AB, Lund, Sweden). Unless otherwise stated, bars in figures represent mean values, ***p<0.001, **p<0.01, *p<0.05, n.s. not significant.

## Results

### Liver tissue specimens and histological grading

We have characterized non-parenchymal intrahepatic cells of immune as well as non-immune cell origin in PSC-patients and controls. The access to clinical material, collected during 2009–2012, enabled us to perform a semi-quantitative, comparative analysis with enumeration of several different cell subsets. The clinical characteristics and histological scores of the patients are presented in Table 1. The median age of the PSC-patients was 36 years (range 19–68), 10 (59%) were male and 7 (41%) were female. The mean MELD score was 13 (range 3–38), 4 of the PSC-patients were assessed histologically as having a mild disease and 13 as having a severe disease.

### IHC characterization and quantitative image analysis of non-parenchymal non-immune cells in PSC-patient livers

**Figure 1:**
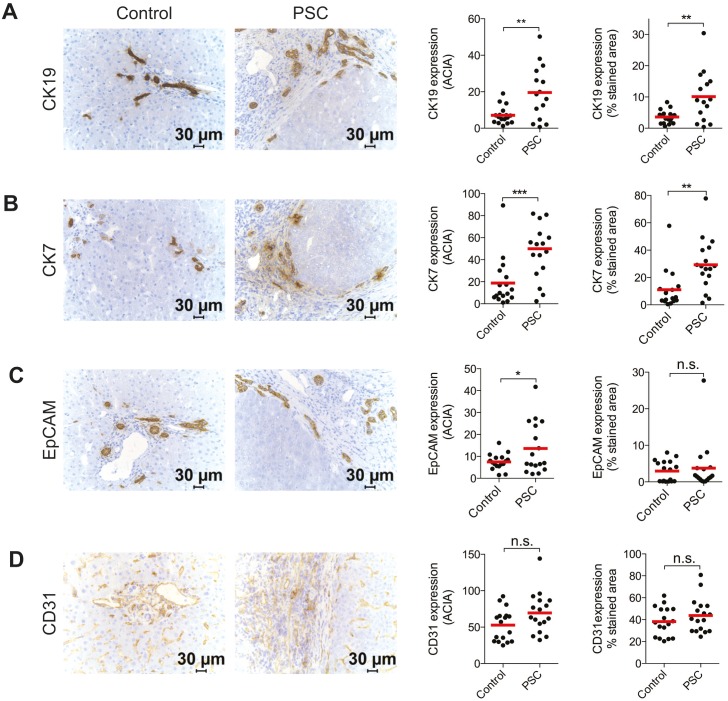
IHC characterization and quantitative image analysis of non-parenchymal non-immune cells of PSC patient livers. Representative immunostaining and quantification analysis of (A) CK19, (B) CK7, (C) EpCAM, (D) CD31 expression in controls (n = 14–17) and PSC patients (n = 14–17). Samples were counterstained with hematoxylin and dark brown indicate immunoreaction product. Quantification was performed to calculate mean intensity of the stainings (ACIA) and frequency of expression (percentage of stained area out of total area).

Antibodies to Cytokeratin 7 (CK7), Cytokeratin 19 (CK19), and Epithelial Cell Adhesion Molecule (EpCAM) were used to detect biliary epithelial cells by immunohistochemistry. For detection of endothelial cells, an antibody against CD31 was used. As expected, expression of CK7, CK19, and EpCAM was significantly increased in PSC-patients compared to controls (Fig. 1A–C). In contrast to the increased protein expression of CK19, only a trend towards increased *CK19* mRNA was noted in the patients compared to the controls (Fig. S1, p = 0.07). Intensity and frequency of expression of CD31 (Fig. 1D) did not differ significantly between PSC-patients and controls. In summary, the results are in line with earlier studies and show that PSC patients have significantly higher numbers of cholangiocytes compared to controls.

### T cells infiltrate PSC-patient livers

**Figure 2:**
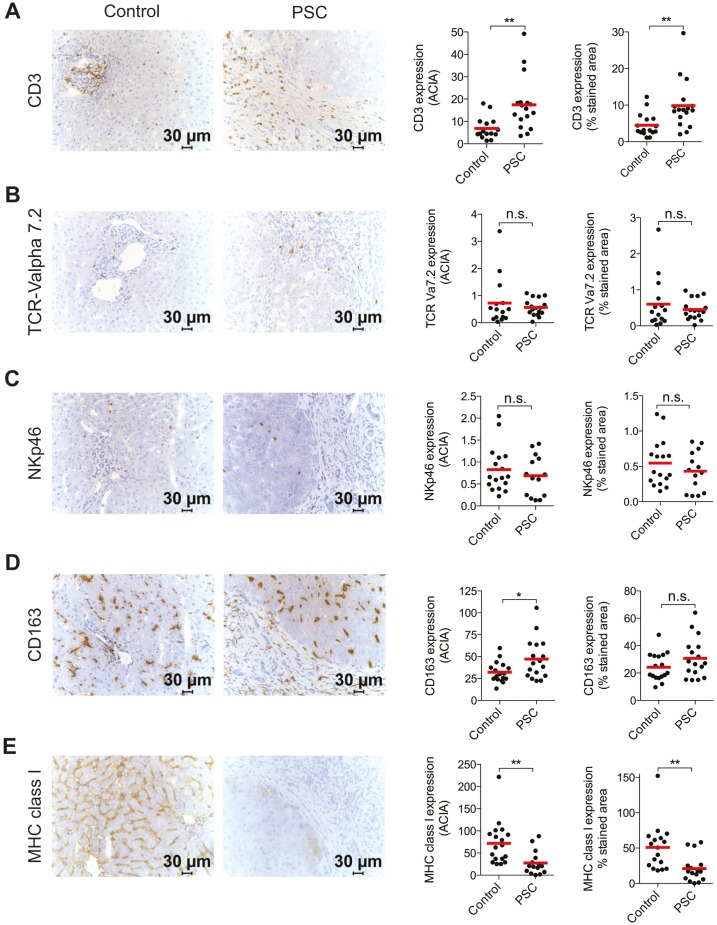
T cells infiltrate PSC-patient livers. Representative immunostaining and quantification analysis of (A) CD3, (B) TCR-Vα7.2, (C) NKp46, (D) CD163, and (E) MHC class I expression in controls (n = 14–17) and PSC patients (n = 14–17). Samples were counterstained with hematoxylin and dark brown indicate immunoreaction product. Quantification was performed to calculate mean intensity of the stainings (ACIA) and frequency of expression (percentage of stained area out of total area).

Liver-infiltrating T cells have been implicated in the pathogenesis of PSC, however little information is available about changes in other intrahepatic immune cell populations during PSC disease. Here, immunostainings were performed to enumerate T cells (CD3), mucosal-associated invariant T (MAIT) cells (TCR-Vα7.2), NK cells (NKp46), Kupffer cells (CD163), and MHC class I (HLA-A, B, C) expressing cells respectively in PSC-patients and controls. Significantly higher expression of CD3 were seen in PSC-patients compared to controls, apparent by both increased ACIA-value and frequency of expression (Fig. 2A), whereas no difference could be seen for TCR-Vα7.2 (Fig. 2B) or NKp46 expression (Fig. 2C). Also *CD3* mRNA was significantly increased in the patients whereas no difference was seen for *NKp46* as compared to controls (Fig. S1). Furthermore, CD163 was more strongly stained in PSC patients compared to controls (Fig. 2D). Next, we evaluated expression of MHC class I in the liver parenchyma, an important regulator of both T- and NK-cell function. Interestingly, both ACIA and frequency of expression was significantly reduced in the PSC-patients (Fig. 2E). In contrast to the loss of total MHC class I protein, no differences in *HLA-A* and *HLA-B* mRNA could be noted comparing the two groups (Fig. S1). Taken together, characterization of innate and adaptive immune cell subsets showed that PSC-patients have significantly higher expression of CD3+ T cells compared to controls, whereas presence of MAIT cells was not equally increased. Furthermore, PSC-patients had a significantly lower expression of MHC class I compared to the controls.

### Low expression of Caldesmon in PSC-patients correlates with higher numbers of T cells and more extensive bile duct proliferation

**Figure 3:**
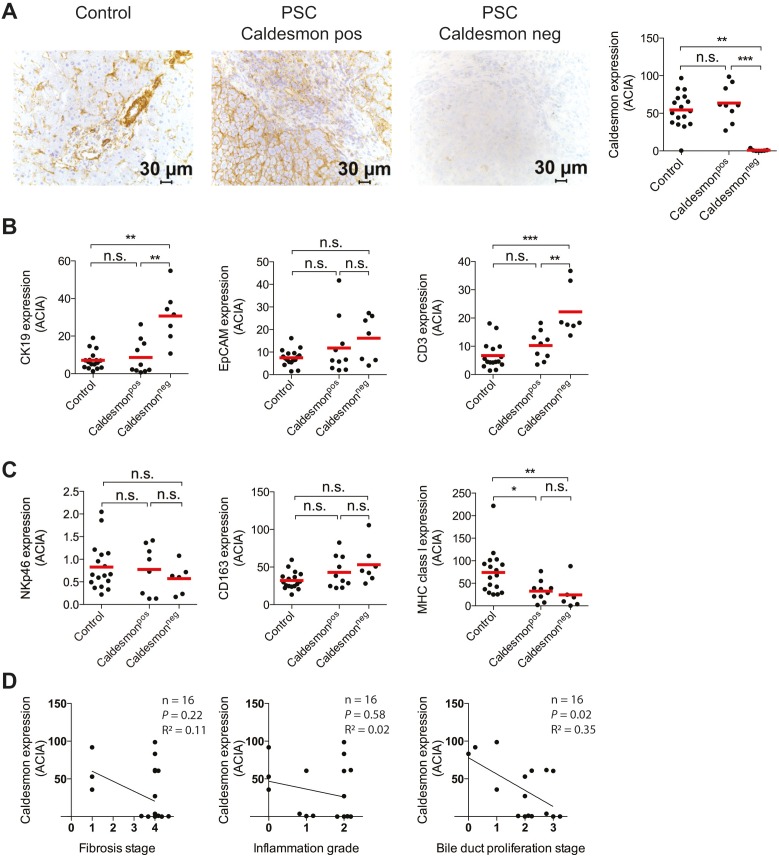
Low expression of Caldesmon in a distinct group of PSC-patients correlates with higher numbers of T cells and more bile duct proliferation. (A) Representative Caldesmon stainings of one control and two PSC-patients (left) and image analysis of Caldesmon expression dividing PSC-patients into groups of high (pos) and low expression (neg) (right). (B and C) Quantification with image analysis of CK19, EpCAM, CD3, NKp46, CD163, and MHC class I expression in controls and PSC patients with pos or neg expression of Caldesmon. (D) Linear regression analysis for Caldesmon and histological parameters of liver disease for the PSC-patients.

Smooth muscle cells may contribute to the repair mechanisms leading to fibrosis. Immunostaining and enumeration was therefore performed for Caldesmon, a protein important for smooth muscle cell function. The IHC analysis of intensity and frequency of Caldesmon expression did not reveal any significant difference between PSC-patients and controls on a group-level (data not shown). Neither could a difference be seen when assessing *Caldesmon* mRNA levels in patients compared to controls (Fig. S1). However, after the IHC analysis, two distinct groups within the PSC cohort were identified: one group with levels similar to that of the controls (Caldesmon^p^°^s^), and a second group with an almost negative expression (Caldesmon^neg^) (Fig. 3A). To analyze this further a comparison between these two groups was made. Significantly higher expression of CD3 was seen in Caldesmon^neg^ compared to Caldesmon^p^°^s^ patients (Fig. 3B). Caldesmon^neg^ patients were further characterized by significantly higher expression of CK19, indicating a more extensive bile duct proliferation, whereas EpCAM was not similarly increased (Fig. 3B). Furthermore, expression of NKp46, CD163, and MHC class I did not differ between the groups (Fig. 3C). When comparing the groups according to histological parameters, a significant decrease of Caldesmon expression was observed with increasing severity of bile duct proliferation (Fig. 3D), whereas no such correlation was seen for fibrosis or inflammation (Fig. 3D). In summary, one distinct population of PSC-patients, characterized by an almost complete loss of the smooth muscle cell marker Caldesmon, have more T cell infiltration and bile duct proliferation, suggesting increased disease activity.

### T cells localize to fibrosis-affected areas in PSC patient-livers

**Figure 4:**
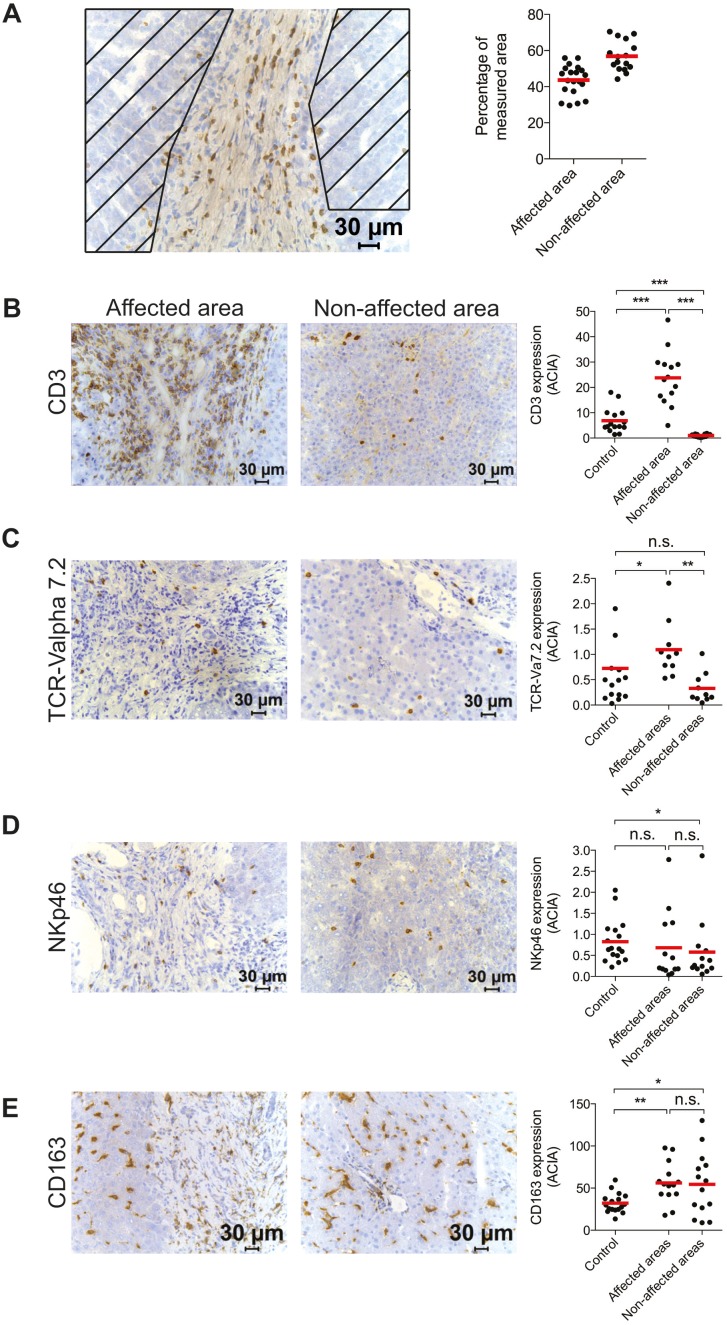
T cells localize to fibrosis-affected areas in PSC patient-livers. (A) Representative photomicrograph for identification of affected and unaffected (striped) areas from one PSC-patient (left) and quantifications of fibrosis affected or non-affected areas in the PSC-patients (right). Immunostainings and quantitative determination of intensity of staining (ACIA) of (B) CD3, (C) TCR-Vα7.2, (D) NKp46, and (E) CD163, in affected and non-affected areas in the patients (CD3, n = 14; TCR-Vα7.2, n = 10; NKp46, n = 13; and CD163, n = 14), and in comparison with total area of controls (n = 14–17).

The analysis of different immune cell subsets revealed a degree of heterogeneity among the PSC-patients, which prompted us to investigate how the cells localize within the tissue, according to fibrosis-affected or unaffected areas. To do this, quantification with image analysis was performed, either excluding or including fibrotic areas. On average, 57% of the measured surface was assessed as unaffected (i.e. non-fibrotic) and 43% as fibrosis-affected (Fig. 4A). T cells were almost exclusively located to fibrotic areas (Fig. 4B). Interestingly, significantly fewer T cells were present in unaffected areas in the PSC patients as compared to the controls (Fig. 4B). A similar pattern was observed for MAIT cells (Fig. 4C). However, with the difference that the MAIT cells were present in equal numbers in the non-fibrotic fields of the patients and in the controls, suggesting less of an accumulation to fibrotic areas as compared to other T cells. NK cells (Fig. 4D) and Kupffer cells (Fig. 4E) were scattered throughout the parenchyma and levels of cells were essentially the same in affected and non-affected areas (Fig. 4D–E). A consequence of the accumulation of CD3+ cells to fibrosis-affected areas in the patients was a redistribution of the cellular composition in non-affected areas. In these areas, the NK cell to T cell ratio and the Kupffer cell to T cell ratio were significantly higher than in the non-PSC controls (Fig. S2). Furthermore, close to equal expression of NKp46 and CD3 was noted in the non-affected areas of PSC-patient livers (Fig. S2).

In conclusion, quantification of different immune cell subsets in fibrosis and non-fibrosis affected areas of PSC-patient samples revealed that T cells and MAIT cells primarily localize to fibrotic fields.

### Principal component analysis of non-parenchymal liver cells in PSC

**Figure 5:**
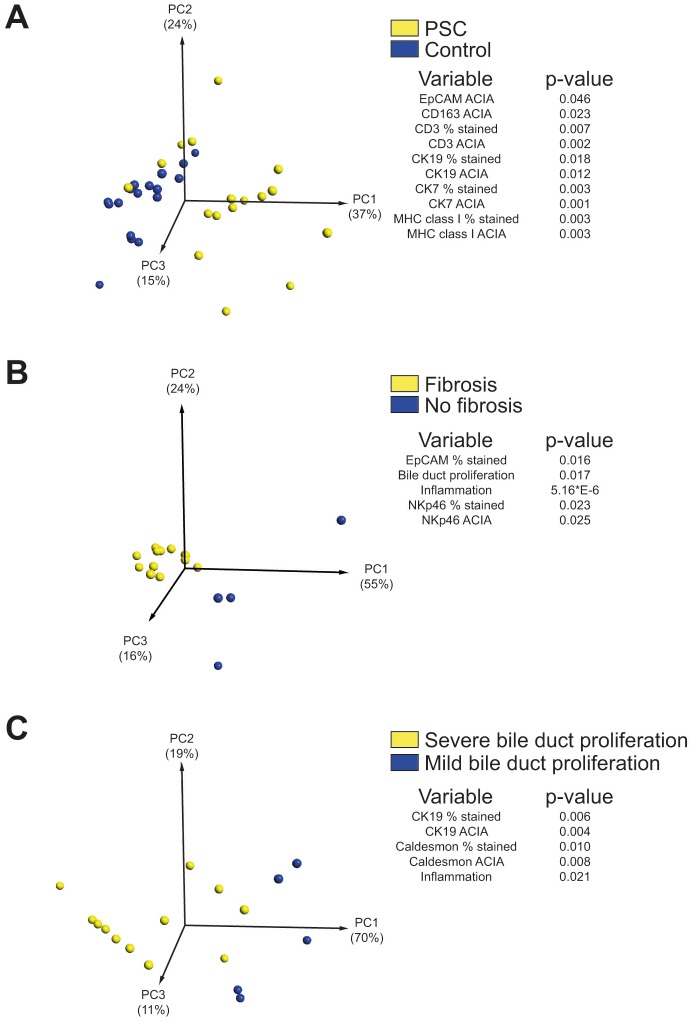
Principal component analysis of non-parenchymal liver cells in PSC. (A) PCA plot showing the parameters that most strongly separate PSC-patients (n = 17) from controls (n = 17) out of totally 20 measured parameters. (B) Comparison of parameters separating PSC-patients with high stage fibrosis (n = 13) from patients with low stage fibrosis (n = 4) from a total of 23 measured parameters. (C) Comparison of parameters that discriminate PSC-patients with severe bile duct proliferation (n = 12) from patients with low bile duct proliferation (n = 5) from a total of 23 measured parameters.

Having analyzed a comprehensive material of immunohistochemical data from histologically assessed PSC-patient samples including a total of 23 unique parameters, we next wanted to identify parameters that separated patients from controls, and further, within the PSC group, identify patterns associated with disease severity. Principal component analysis (PCA) was performed with the 20 measured parameters, excluding histological scores (fibrosis, inflammation, and bile duct proliferation), comparing PSC-patients with controls. As expected, the biliary epithelial cell markers EpCAM, CK7, and CK19 were all markers separating PSC-patients from controls (Fig. 5A). Furthermore, expression of CD3, CD163, and MHC class I significantly differed between patients and controls (Fig. 5A).

In a second set of analysis, 23 measured parameters within the PSC group, including histological scores, were compared to identify phenotypic markers that differentiated patients with respect to severity of fibrosis or bile duct proliferation (Fig. 5B–C). EpCAM expression (p = 0.016) was identified as a factor that separated PSC-patients with high stage from low stage of fibrosis, whereas no such association was found for the other biliary markers CK7 and CK19. Both frequency of expression and ACIA-value of NKp46 were also identified as factors that significantly separated PSC-patients according to fibrosis severity (p = 0.023 and p = 0.025). In addition to EpCAM and NKp46 expression, the histological parameters inflammation and bile duct proliferation were additional factors separating PSC-patients based on fibrosis stage (Fig. 5B, p = 5*10^−6^ and p = 0.017). Expression of Caldesmon, CK19, and the degree of inflammation were components that differentiated patients with severe- from mild bile duct proliferation (Fig. 5C). In summary, the PCA analysis revealed that numbers of bile duct cells, T cells, Kupffer cells, and MHC class I expression significantly separated PSC-patients and controls. Further comparisons, including histological scores, identified EpCAM and NKp46 as phenotypic markers associated with severity of fibrosis, and Caldesmon and CK19 as markers correlated with bile duct proliferation stage.

## Discussion

In this study, we have characterized and quantified intrahepatic non-parenchymal cells in PSC with the aim to find distinct phenotypes and investigate whether these associate with disease severity. As expected, significant infiltration of CD3^+^ T cells, preferably localizing to fibrotic areas, was detected in PSC-patients compared to controls. However, this infiltration was specific to certain subsets of T cells since TCR-Vα7.2^+^ MAIT cells, normally present at high frequencies in the liver [13], were not similarly increased in the PSC-patients. Finally, a distinct group of PSC-patients, characterized by having an almost complete loss of Caldesmon expression, had more T cell infiltration, reduced MHC class I expression, and more extensive bile duct proliferation. Our data show diversity among PSC-patients and reveals phenotypic patterns that correlate with disease severity.

Caldesmon is a protein associated with cytoskeletal structures and appears to participate in many different biological processes, such as cell division, migration, adhesion and apoptosis [14]–[16]. To our knowledge, no previous study has investigated a link between reduced expression of Caldesmon and PSC disease severity. Why certain PSC-patients lose Caldesmon expression remains elusive. The observations that these patients had increased T cell infiltration and more bile duct proliferation, however, suggest a more active liver disease compared to the group with normal expression. Reduced Caldesmon expression might be a cause of more inflammation and T cell infiltration, or a result of other cellular processes, such as wound healing or fibrosis, which in turn may lead to activation of T cells and increased bile duct proliferation. However, our data from the PCA analysis, which identified Caldesmon as a factor distinguishing PSC-patients with mild from severe bile duct proliferation suggests that decreased Caldesmon expression might be a response to cellular reactions leading to biliary proliferation. TGF-β has earlier been identified as a factor that can reduce Caldesmon expression in certain pathological conditions [17]. Caldesmon contributes to contractile properties in various organs, but it is unclear what sort of physiological effect the reduced expression in the liver might have. The molecular mechanisms underlying the reduced expression observed in some PSC-patients, and the role of this in PSC pathogenesis, needs to be further investigated.

As expected, a significant infiltration of CD3^+^ T cells, preferably localizing to fibrosis-affected areas, was detected in PSC livers compared to controls. Previous immunohistochemical studies have revealed that the inflammatory infiltrate in PSC livers consists primarily of CD8^+^ T cells, concentrated around the portal tracts [18]. Further characterization studies showed that the inflammatory infiltrates in PSC livers are non-activated memory T cells that express the gut-homing integrin alpha4beta7 [10], [19]. It has been suggested that gut-restricted T cells, once activated in the intestine, migrate to the liver in response to up-regulated molecules on sinusoidal endothelial cells [10], [11], [20]. Further support of this theory came from a murine experimental model where CD8^+^ T cells activated in the gut caused immune-mediated cholangitis in an antigen-dependent manner [9]. The results from our study are in line with previous results and show that T cells are highly increased in numbers in PSC-patients and are likely to play a significant role in the pathogenesis of PSC.

MAIT cells are recently described invariant T cells enriched in the human liver with the capacity to recognize bacterial antigens and produce Th1/Th17 cytokines [13]. Interestingly, PSC-patients have increased amounts of bacterial and fungal products in bile compared to healthy controls and PBC patients. Furthermore, bacteria from PSC patients bile fluid have been shown to generate high frequencies of peripheral blood Th17 and Th1/Th17 cells [8]. We observed MAIT cells to localize preferentially to fibrosis-affected areas in PSC-patients. The levels overall, however, were approximately the same in PSC-patients as compared to controls and the accumulation to fibrotic areas was not of the same magnitude as for total CD3+ T cells. Thus, it is currently unclear if MAIT cells contribute to PSC-pathogenesis and future studies focusing on the function of MAIT cells in PSC will be of importance to determine their potential role in the pathogenesis.

Except for a report in the early 1990ies [21], the presence and possible role of NK cells has previously not been investigated in PSC. In this study we could not detect any significant difference in numbers of NK cells between PSC patients and controls. However, patients with a low stage of fibrosis were observed to have higher levels of NK cells as revealed by the PCA-analysis. Interestingly, NK cells did not localize preferentially to areas of fibrosis, instead they were scattered throughout the parenchyma. Several studies, mainly performed in murine models, have suggested NK cells to have anti-fibrotic properties. Furthermore, human NK cells, isolated from HCV-infected liver samples, were shown to efficiently induce apoptosis of activated HSCs [22]. These reports are consistent with our finding that PSC-patients with less fibrosis have more NK cells.

The genetic profile of PSC is similar to that of typical HLA-associated diseases. Genetic variations that affect the peptide binding function of HLA class I- and II molecules are considered to have a central role in PSC. However, few studies have examined MHC class I expression at the protein level in PSC. We show that MHC class I expression is significantly reduced in PSC-patients compared to controls. This was further confirmed with the PCA analysis, which identified MHC class I as a factor that distinguished PSC-patients from controls. It has previously been reported that ursodeoxycholic acid (UDCA) treatment of PBC-patients results in reduced MHC class I expression by hepatocytes [23]. Further studies of the immunological effects of UDCA revealed a connection between levels of IFN-γ and expression of MHC class I [24]. IFN-γ, known to up-regulate MHC class I expression, was significantly increased in PBC patients compared to controls. However, UDCA treatment led to decreased levels of IFN-γ and consequently lower expression of MHC class I [24]. All PSC patients included in our study received UDCA treatment, and corroborating previous reports, PSC patients had lower expression of MHC class I compared to controls.

A limitation of the current study is the age difference between the non-PSC and the PSC group. Thus, it is plausible that the intrahepatic non-parenchymal cell compartment changes with ageing. However, when evaluating this for the samples used in the study, we could not detect any significant correlations between age and expression of a majority of the investigated markers. The only significant correlation present was a negative correlation between expression of CD31 and age. Thus, we find it less likely that the age-difference between the two groups has an influence on the results. Furthermore, the non-PSC group was heterogeneous with four samples from deceased transplant donors and 13 from patients with tumor metastasis. However, no significant differences were detected when comparing expression of the investigated markers between the deceased donors and the metastasis patients

Another limitation of the current study is that the majority of the PSC-patient samples were from patients with end-stage disease. Only four patients had mild disease, and due to this relatively low number, correlations with clinical parameters should be interpreted with caution. Additionally, findings presented here might better portray late events occurring in the liver rather than initial changes in the early stage of disease. Future studies focusing on replication of the current results are warranted as well as studies focusing on patients with mild disease. Unfortunately, getting access to such material is challenging and needle biopsy samples may not be representative of the entire liver as such. Additionally, because of the limited sample size, associations might have been lost due to low power.

In this study, samples from PSC patients were compared to control livers not allowing us to determine if observed changes were specific for PSC or also present in other chronic liver diseases. The loss of Caldesmon with the accompanying accumulation of T cells in a subgroup of PSC-patients should be reevaluated in other chronic liver diseases as well as the finding that only T cells and MAITs but not NK cells and Kupffer cells accumulated in fibrotic areas. Of note, the current study is also descriptive in nature and future efforts should focus in studying the biology of fibrosis accumulating immune cells as well as on the mechanism behind and consequences of the loss of Caldesmon in PSC-patients.

Taken together, the aim of this study was to characterize and localize intrahepatic cells of both immune as well as non-immune cell origin in PSC livers in order to identify distinct phenotypes, and secondly to investigate if these were associated with disease severity. Our data reveal that there is heterogeneity in the PSC group, and that individuals with certain phenotypic patterns have higher numbers of T cells and more extensive bile duct proliferation, indicating an increased disease severity.

## Supporting Information

Figure S1 **mRNA expression of immune and non-immune markers in control and PSC-patient livers.** Total mRNA was isolated from control (n = 17) and PSC-patient (n = 17) livers and mRNA expression of *CK19*, *Caldesmon*, *CD3*, *NKp46*, *HLA-A*, and *HLA-B* was assessed. Values are presented relative to the expression of the housekeeping gene *Cyclophilin A*.
(TIF)

Figure S2 **Comparison of immune cell to T cell ratios in controls and PSC patients.** The ratios of ACIA (mean intensity of staining) of the different immune cells to ACIA of CD3 expression were calculated and compared between controls (n = 10–17) and PSC patients (n = 10–17) and within the PSC group between affected and non-affected areas. (A) TCR-Vα7.2 expression to CD3 expression, (B) NKp46 expression to CD3 expression, and (C) CD163 expression to CD3 expression.
(TIF)
