# Supplementary figures and images for: In Situ Characterization of Intrahepatic Non-Parenchymal Cells in PSC Reveals Phenotypic Patterns Associated with Disease Severity
Source: PLoS One. 2014 Aug 20;9(8):e105375. doi: 10.1371/journal.pone.0105375 (PMC4139378; doi:10.1371/journal.pone.0105375)

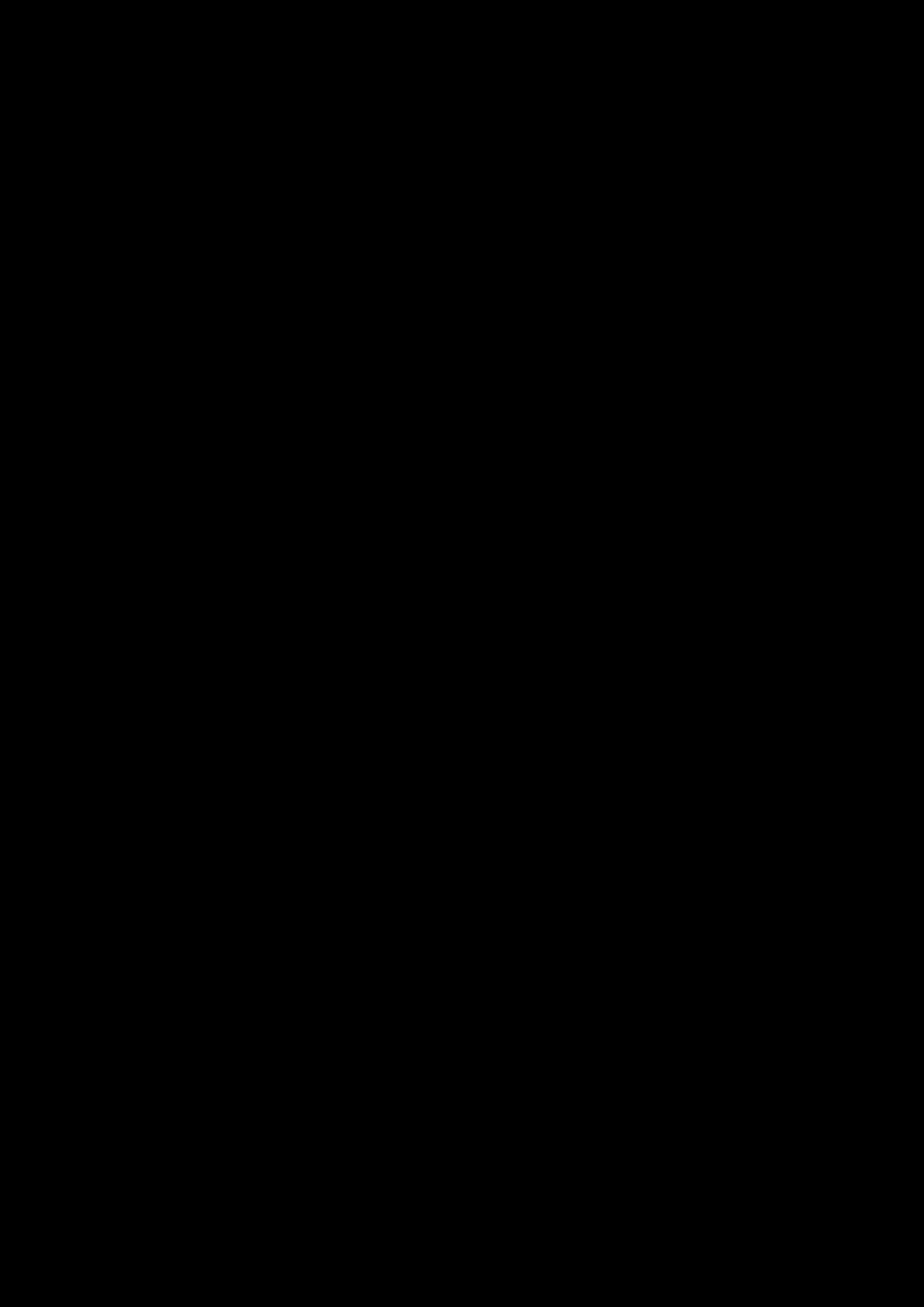

Supplement: Figure S1 — mRNA expression of immune and non-immune markers in control and PSC-patient livers. Total mRNA was isolated from control (n = 17) and PSC-patient (n = 17) livers and mRNA expression of CK19, Caldesmon, CD3, NKp46, HLA-A, and HLA-B was assessed. Values are presented relative to the expression of the housekeeping gene Cyclophilin A. (TIF) [file pone.0105375.s001.tif]

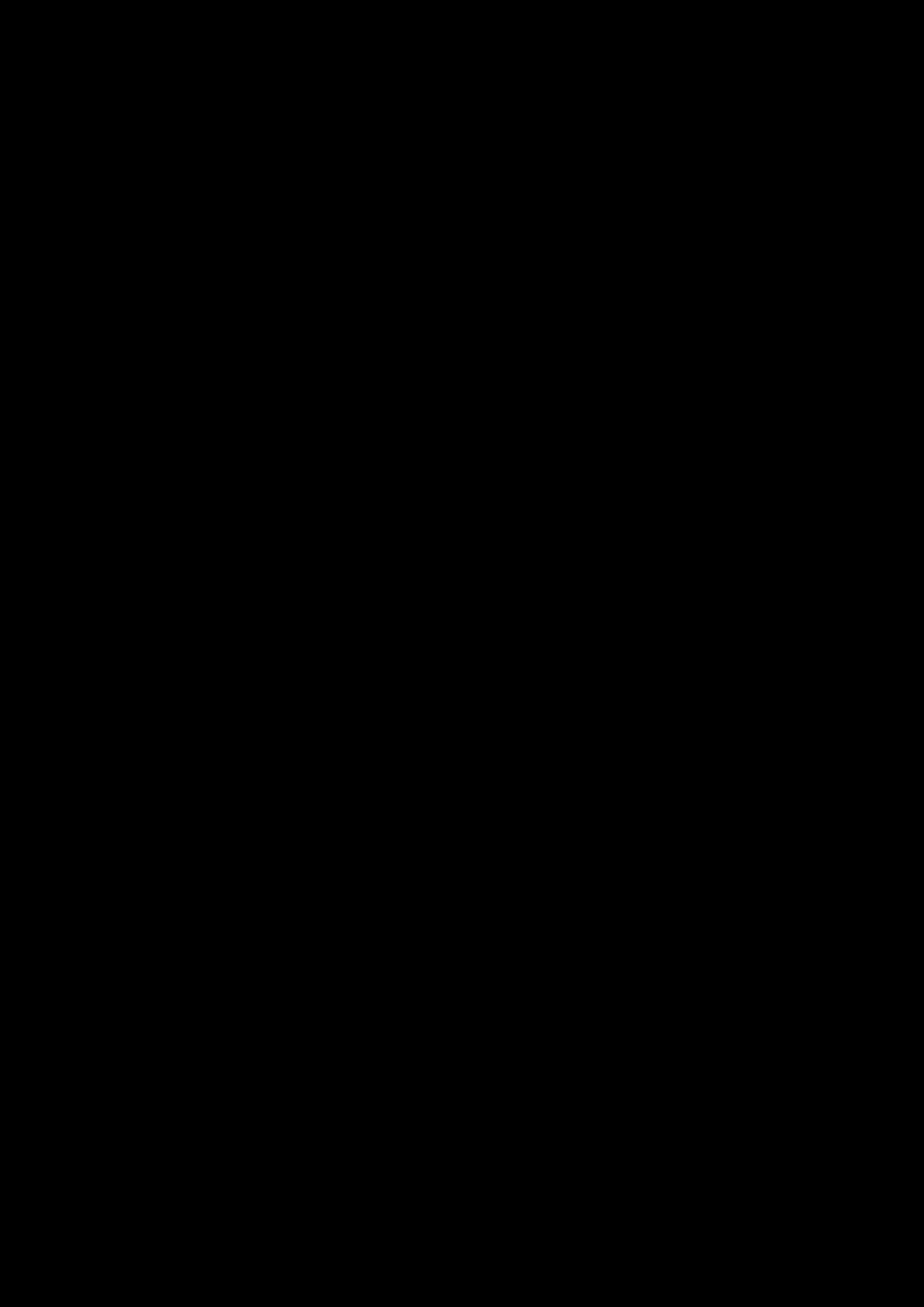

Supplement: Figure S2 — Comparison of immune cell to T cell ratios in controls and PSC patients. The ratios of ACIA (mean intensity of staining) of the different immune cells to ACIA of CD3 expression were calculated and compared between controls (n = 10–17) and PSC patients (n = 10–17) and within the PSC group between affected and non-affected areas. (A) TCR-Vα7.2 expression to CD3 expression, (B) NKp46 expression to CD3 expression, and (C) CD163 expression to CD3 expression. (TIF) [file pone.0105375.s002.tif]
